# Novel mutations in *MERTK* associated with childhood onset rod-cone dystrophy

**Published:** 2010-03-09

**Authors:** Donna S. Mackay, Robert H. Henderson, Panagiotis I. Sergouniotis, Zheng Li, Phillip Moradi, Graham E. Holder, Naushin Waseem, Shomi S. Bhattacharya, Mohammed A. Aldahmesh, Fowzan S. Alkuraya, Brian Meyer, Andrew R. Webster, Anthony T. Moore

**Affiliations:** 1 Institute of Ophthalmology, London, UK; 2 Moorfields Eye Hospital, London, UK; 3 Department of Genetics, King Faisal Specialist Hospital & Research Center, Riyadh, Saudi Arabia

## Abstract

**Purpose:**

To report the clinical phenotype in patients with a retinal dystrophy associated with novel mutations in the MER tyrosine kinase (*MERTK)* gene.

**Methods:**

A consanguineous family of Middle Eastern origin was identified, and affected members underwent a full clinical evaluation. Linkage analysis was performed using the Affymetrix 50K chip. Regions of homozygosity were identified. The positional candidate genes protocadherin 21 (*PCDH21)*, retinal G protein-coupled receptor (*RGR)*, and *MERTK* were polymerase chain reaction (PCR) amplified and sequenced. Long-range PCR was performed to characterize the deletion. Two hundred and ninety-two probands with autosomal recessive, childhood onset, retinal dystrophies were analyzed using the Asper Ophthalmics Leber congenital amaurosis chip to screen for known *MERTK* mutations.

**Results:**

Analysis of a 50K-Affymetrix whole genome scan identified three regions of homozygosity on chromosomes 2 and 10. Screening of the candidate gene *MERTK* showed a possible deletion of exon 8. Long-range PCR identified a ~9 kb deletion within *MERTK* that removes exon 8. Screening of DNA from a panel of Saudi Arabian patients with autosomal recessive retinitis pigmentosa identified a second consanguineous family with the same mutation. One patient with a known *MERTK* mutation (p.R651X) was identified using the Asper Ophthalmics Leber congenital amaurosis chip. Further screening of the gene identified a second novel splice site mutation in intron 1. The phenotype associated with these identified *MERTK* mutations is of a childhood onset rod–cone dystrophy with early macular atrophy. The optical coherence tomography (OCT) appearance is distinctive with evidence of debris beneath the sensory retina.

**Conclusions:**

Mutations in *MERTK* are a rare cause of retinal dystrophy. Non homologous recombination between Alu Y repeats near or within disease genes may be an important cause of retinal dystrophies.

## Introduction

Retinitis pigmentosa (RP) describes a group of disorders with progressive degeneration of rod and cone photoreceptors in a rod–cone pattern of dysfunction. RP has a prevalence of 1 in 3,500 [1]. It is genetically and phenotypically heterogeneous, with all three forms of Mendelian inheritance having been reported. Autosomal recessive RP (ARRP) is the most common form, accounting for more than half of all cases of RP [2]. To date, mutations in 25 genes have been associated with ARRP, with a further four mapped loci (RetNet).

MER tyrosine kinase *(MERTK),* a member of the Axl/Mer/Tyro3 receptor tyrosine kinase family required for phagocytosis and expressed in the retinal pigment epithelium (RPE) [3], was first implicated in retinal degeneration in 2000 when a deletion in the gene was identified in the Royal College of Surgeons (RCS) rat [4]. Gal et al. [5] subsequently identified three different mutations in patients with recessive retinal dystrophies. However, in comparison to other genes involved in retinal dystrophies, the reporting of new mutations in *MERTK* has been slow, with only a further five mutations having been described since the initial study [6-11].

The present report describes the clinical phenotype associated with two novel mutations in *MERTK*.

## Methods

### Clinical investigations

Two 10 ml EDTA tubes of peripheral venous blood were drawn from all available family members, including both affected and unaffected individuals, and samples were frozen. DNA was extracted using the Nucleon™ BACC-2 genomic DNA kit (GE Healthcare Life Sciences, Buckinghamshire, UK). Following cell lysis, deproteinization was performed using sodium perchlorate. DNA extraction was achieved with chloroform and Nucleon™ resin before DNA recovery and washing.

All patients involved in this study provided informed consent as part of a research project approved by Moorfields Eye hospital and King Faisal Specialist Hospital research ethics committee, and all investigations were conducted in accordance with the principles of the Declaration of Helsinki. Clinical evaluation including slit-lamp examination, assessment of visual acuity, color vision, and perimetry were performed. Color vision was evaluated using the Hardy-Rand-Rittler (HRR) plates (American Optical Company, New York, NY) and the 24-plate Ishihara color vision test (Kanehara and Co. Ltd, Tokyo, Japan). Patients underwent retinal imaging, including fundus autofluorescence imaging (FAF), with confocal scanning laser ophthalmoscopy (Heidelberg Retina Angiograph 2 [HRA2]; Heidelberg Retina Angiograph OCT [HRA-OCT]; Heidelberg Engineering, Heidelberg, Germany). Full-field and pattern electroretinography (ERG and PERG) were performed with gold foil recording electrodes incorporating the International Society for Clinical Electrophysiology of Vision (ISCEV) Standards [12,13]

### Apex chip

A genomic DNA sample from 292 unrelated affected patients with Leber congenital amaurosis (LCA) or childhood onset RP was sent to Asper Ophthalmics Ltd for analysis, as described previously [14,15].

### Autozygosity mapping

Genome-wide linkage scan were performed on family A to identify regions of autozygosity (where both alleles are identical by descent and are copies of a common ancestral gene). DNA samples from all affected family members were genotyped using the Affymetrix human GeneChip® Mapping 50K XbaI Array (Affymetrix, Santa Clara, CA). The detailed methodology for genotyping using the GeneChip® array has been previously described [16]. Briefly, 250 ng of genomic DNA was digested with XbaI (New England Biolabs, Ipswich, MA) for 2 h at 37 °C, ligated with XbaI adaptors using T4 DNA ligase (New England Biolabs, Hitchin, Herts, UK). The ligation reaction was diluted in 1:4 (v/v) with molecular grade water (Sigma-Aldrich, St. Louis, MO) to 100 µl. Ten µl of the diluted ligation mix was used to set up selection by PCR (Fragment Selection by PCR) in triplicates. The pooled PCR products were purified using QIAGEN MinElute 96 UF plate (Qiagen, Duesseldorf, Germany). The concentration of PCR products was quantified using ND-1000 Nanodrop spectrometry (Thermo Fisher Scientific, Waltham, MA). Purified PCR product (90 ng) was fragmented with 0.25 units DNaseI ('Fragmentation Reagent'; Affymetrix), labeled with 'Labeling Regent' (Affymetrix). The labeling reaction (70 µl) was mixed with 190 μl hybridization reagent and denatured at 99 °C for 10 min (all as detailed in the Affymetrix GeneChip Mapping 100K Assay Manual). Finally the denatured hybridization mixture was injected into Affymetrix Human Mapping XbaI chips and incubated at 48 °C for 16 h, followed by automatic washing and staining in a Fluidics Station 450, and scanned by using the GeneChip® Scanner 3000 7G (Affymetrix). Genotypes for single nucleotide polymorphisms (SNPs) were called by the GeneChip DNA Analysis Software (GDAS version 3.0, Affymetrix). A macro was written in Visual Basic within the Microsoft Excel (Microsoft, Redmond, WA) program to detect genomic regions showing regions of shared haplotype.

### Mutation screening

Primers to amplify the coding exons and the intron–exon boundaries of *MERTK*, protocadherin 21 (*PCDH21*), and retinal protein-coupled receptor (*RGR*) were used as previously described [10,17,18]. All PCR were performed in a total volume of 30 μl containing 200 μM deoxynucleotide triphosphate (dNTPs; VH Bio, Gateshead, UK), 20 μM of each primer, 1× reaction buffer (10× PCR buffer: 600 mM Tris-sulfate [pH 9.1], 180 mM ammonium sulfate, 15 mM magnesium sulfate; VH Bio) with 1 unit of Moltaq (VH Bio) and 100 ng of DNA. PCR was performed on a PTC200 DNA engine thermal cycler (Bio-Rad, Hemel Hempstead, UK).

PCR products were visualized on a 2% agarose gel containing 0.05% ethidium bromide. The products were cleaned using multiscreen PCR filter plates (Catalogue no LSKMPCR10; Millipore, Watford, UK) before sequencing. PCR products were sequenced directly using ABI Prism Big Dye Terminator kit (ABI Ltd, Warrington, Cheshire, UK; version 3.1) in a 10-μl reaction. Samples were purified using the Montage cleanup kit (catalog no LSK509624; Millipore) before being run on an ABI Applied Biosystems 3730 DNA sequencer.

Long-range PCR was used to determine the size of the deletion. PCR primers were designed ranging from intron 7 (7F2, 5′-TGC TGA TAT TCT AGT AGC CAA GTG G-3′) of the *MERTK* gene to intron 8 (8R5, 5′-ACA TTT CTC TGA CAT GAG GTG GTC TG-3′). A total of 12.5 μl of Extensor Hi-Fidelity PCR Master Mix (Abgene, Epson, UK) was added to a 12.5-μl mixture containing water, 250 ng of template DNA, and 200 nM of each primer. Thermal cycling conditions for long-range PCR were an initial denaturation of 2 min at 94 °C was followed by 10 cycles of 94 °C for 10 s, 60 °C for 30 s and 68 °C for 8 min. This was followed by 20 cycles of 94 °C for 10 s, 60 °C for 30 s and 68 °C for 8 min adding 10 s on every cycle.

## Results

### Case report

**Figure 1:**
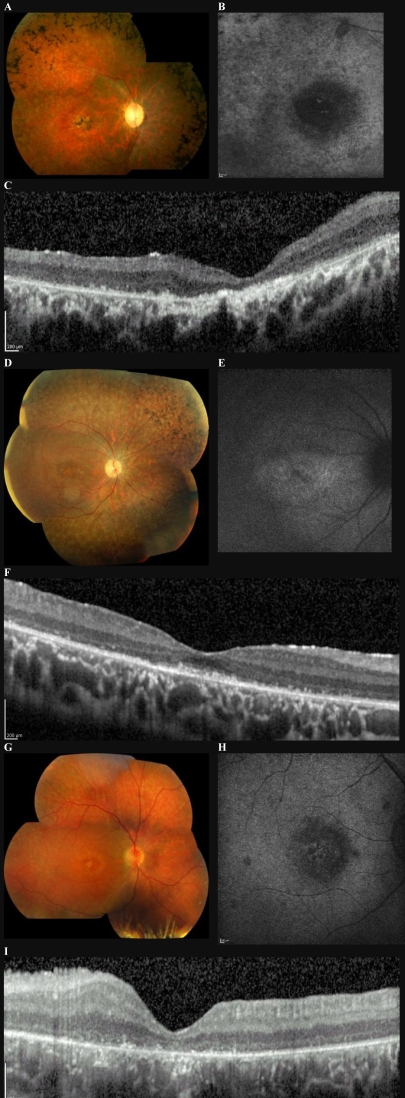
Color fundus composite showing fundus autofluorescence (FAF) and spectral domain OCT (SD-OCT) for patients 1, 2, and 3. Patient 1 (Family A, Individual IV:2) at age 28: fundus image (**A**) shows well circumscribed macular atrophy, vascular attenuation, disc pallor, and peripheral pigment migration. FAF (**B**) using high gain demonstrates the absence of macular autofluorescence. SD-OCT (**C**) reveals thinning of the photoreceptor layer and wrinkling of the outer limiting membrane, with multiple high reflectance bodies visible in residual outer nuclear layer. Patient 2 (Family A, Individual IV: 5) at age 12: fundus image (**D**) shows early macular atrophy and peripheral pigment migration. FAF (**E**) reveals limited parafoveal hyperfluorescence with low total autofluorescence. SD-OCT (**F**) reveals thinning of the photoreceptor layer, with discrete hyper-reflective bodies below the outer limiting membrane. Fundus composite (**G**) of patient 3 at age 23 shows foveal and parafoveal yellow discoloration but minimal peripheral pigmentation. FAF (**H**) demonstrates hypofluorescence at the fovea and SD-OCT (**I**) shows thinning of the photoreceptor layer and high reflectance bodies.

Subject 1. The proband of family A (individual IV:2), a 26-year-old male of Middle Eastern origin, was diagnosed with rod–cone dystrophy at the age of 16. There was no significant history of systemic disease. He first noticed difficulties with night vision and visual field loss at the age of 9 years; reduced central vision was noted at 13 years. When examined at age 26, visual acuity was 1.78 logarithm of the minimum angle of resolution (LogMAR) in each eye. He was unable to recognize any of the plates of the HRR color vision test. Fundus examination revealed pale optic discs, attenuated retinal vessels, macular atrophy, and peripheral retinal atrophy with intraretinal pigmentary deposition extending into the posterior pole in each eye (Figure 1A). FAF demonstrated a large central area of decreased autofluorescence along with more widespread peripheral areas of reduced signal in both eyes (Figure 1B). Spectralis spectral-domain OCT (SD-OCT) imaging (Figure 1C) revealed disruption of the normal laminar arrangement at the photoreceptor/RPE interface and loss of the outer limiting membrane. Discrete hyper-reflective bodies were visible within the outer nuclear layer. Significant loss of retinal volume and foveal atrophy could be observed. Mean retinal thickness of the central Early Treatment Diabetic Retinopathy Study (ETDRS) subfield (CSF) was 182 µm for the right and 165 µm for the left eyes (normal value 270±22.5 µm [19]), and central point thickness (CPT) was 70 µm for the right and 64 µm for the left eyes (normal value 227.3±23.2 µm [19]).

Subject 2. This 8-year-old boy (IV:5), the younger brother of subject 1, was diagnosed with rod–cone dystrophy at the age of 8 years. Parents reported him having difficulty seeing in the dark from an early age. His best corrected visual acuities were 0.32 LogMAR in each eye. He had a low myopic refractive error (−2.00 OU) and mild generalized dyschomatopsia on testing with the HRR color vision test. Visual fields were reduced to 20–30° in each eye. Fundus examination revealed mild macular RPE changes in a “bull’s-eye” pattern, with retinal atrophy and intraretinal pigmentary deposition in the periphery (Figure 1D). There was some residual macular autofluorescence on FAF (Figure 1E). Spectralis OCT (Figure 1F) revealed disruption of the normal foveal inner segment/outer segment (IS/OS) boundary, and smaller hyper-reflective bodies both at the level of the IS/OS junction and in the outer nuclear layer. There was reduced retinal thickness at the fovea, with a mean CSF retinal thickness of 169 µm for the right and 160 µm for the left eyes (normal value 270 ±22.5 µm [19]). CPT was 138 µm for the right and 130 µm for the left eyes (normal value 227.3±23.2 µm [19]).

Subject 3. This 22-year-old male of Caucasian origin was diagnosed with rod–cone dystrophy at the age of 14. He had a strong family history of red–green color blindness (affecting maternal uncle and grandfather and two cousins) and also a family history of deafness (affecting mother, maternal uncle, and grandmother). There was no consanguinity, no family history of retinal dystrophy, and no history of systemic disease. He had normal hearing. He noticed nyctalopia at age 12 and developed sensitivity to light soon after. At age 22, visual acuities were 0.6 LogMAR in the right and 1.0 LogMAR in the left eyes. He had a mild refractive error (OD +0.25/–1.50x120, OS +0.25/–1.00x95) and very poor color vision. Goldman visual fields performed at age 19 were reported to be full. Fundus examination revealed focal atrophy in the central macula and a mild diffuse abnormality of the RPE at the posterior pole but very little intraretinal pigment. Retinal vessels were thinned (Figure 1G**)**. FAF imaging demonstrated a half-disc-diameter-sized central area of hyperfluorescence, encircled by an area of relatively reduced autofluorescence (Figure 1H). Spectralis OCT revealed significant loss of the photoreceptor layer. Mean CSF retinal thickness was 196 µm for the right and 202 µm for the left eyes (normal value 270±22.5 µm [19]). CPT was less than 130 µm in both eyes (normal value 227.3±23.2 µm [19]). Multiple small hyper-reflective bodies could be observed at the level of the IS/OS junction.

Electroretinography was performed at age 16 and 21. In the initial recordings the full-field rod-specific ERGs were undetectable: to a red flash under dark adaptation there was a clear and delayed cone component but no rod component; bright flash ERGs showed a markedly delayed and subnormal a-wave, with a slightly lower amplitude b-wave, and probably arose from residual cones; cone-derived photopic single flash and flicker ERGs were severely delayed and were subnormal. The findings at aged 21 showed no definite clinically significant deterioration. Pattern ERGs on both occasions showed possible low-amplitude residual signals but suggested severe bilateral macular involvement.

### Genome scan

Results of the Affymetrix 50K genome scan on family A revealed three shared regions of homozygosity. Two regions were found on chromosome 2: 2q12-q21 covering 30 Mb in size, a region containing the candidate gene *MERTK;* and 2p13.2-p11.2 covering 16 Mb. An 8-Mb region on 10q23 contained two candidate genes known to be expressed in the eye, *RGR* and *PCDH21*. All three possible candidate genes were screened for mutations [5,17,18]. No sequence changes segregating with disease were found in *RGR* and *PCDH21*.

When screening *MERTK*, all exons apart from exon 8 amplified, and sequencing showed no disease-causing mutations. Only one SNP was seen in the family; rs13027171 (G>A S475N) was heterozygous in individual III:2. All other members of the family were homozygous for the common G allele. PCRs containing exon 8 did not work for either of the affected siblings but worked in control DNA. New primers situated 500 bp either side of exon 8 were designed to rule out a sequence change where the primers annealed. The PCR worked for all unaffected members of the family but not the affected siblings.

**Figure 2:**
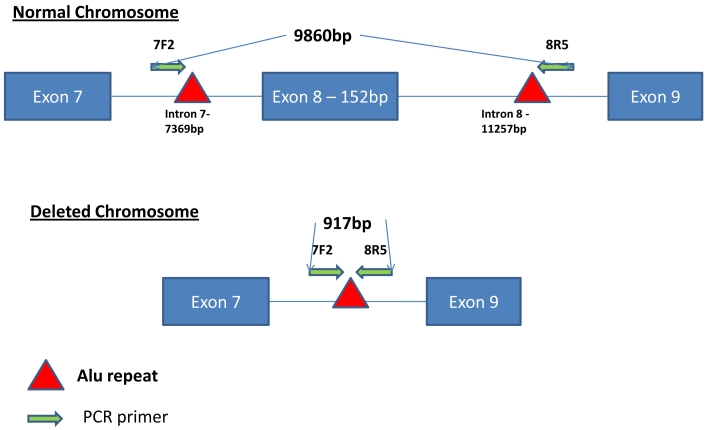
Diagram showing the genomic layout surrounding *MERTK* exon 8 in the normal and deleted chromosomes in Family A. Primers used in the amplification of the deletions are shown by the arrows (labeled 7F2 and 8R5). The position of Alu Y repeats are shown by a red triangle.

**Figure 3:**
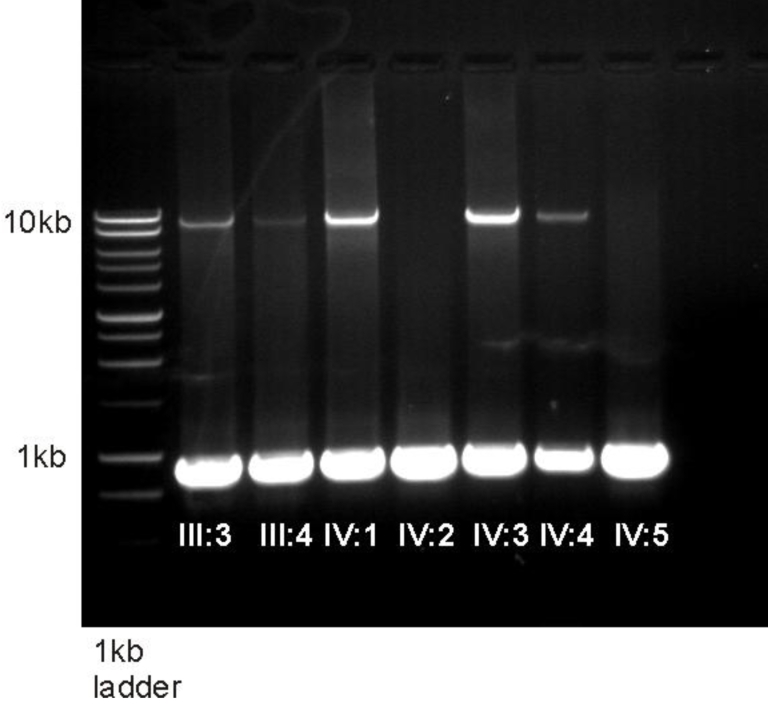
Gel electrophoresis results for long range PCR in family A. Long range PCR (using primers 7F2 and 8R5) showing the normal product of 9.65 kb and the deleted product of 917 bp in Family A. The two affected products (IV:2, IV:5) are missing the upper 9.65-kb band.

**Figure 4:**
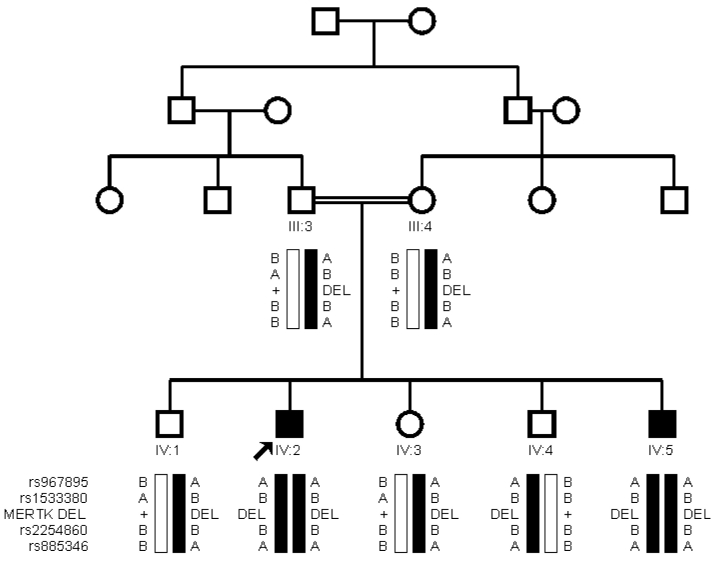
Haplotype analysis of the single nucleotide polymorphism (SNP’s) surrounding the MER protein kinase (*MERTK*) gene in Family A. Haplotyped pedigree of Family A showing the segregation of the deletion with the surrounding SNPs (rs ID numbers to the left of the haplotype) used to map the family to this region. The proband (IV:2, patient 1) is marked by an arrow. The black bar represents the disease haplotype.

A DNA walking PCR-based experiment was designed to cover the area between exon 7 and exon 9 (18,778 bp) to determine if there was a deletion of exon 8. Long-distance PCR between two primers, 7F2 (situated in intron 7) and 8R5 (situated in intron 8), amplified a 9.86-kb product in control samples. When the same region was amplified from the affected siblings, the PCR produced a band of 917 bp instead of the control 9.86 kb, suggesting deletion of exon 8 along with the majority of introns 7 and 8 (Figure 2). Removal of exon 8 is predicted to disrupt the reading frame of *MERTK*, leading to a premature stop codon in exon 9. Both parents and three unaffected siblings amplified both the large and the small fragment (Figure 3). SNP haplotyping of the area surrounding the deletion confirmed that each parent was a carrier of the haplotype containing the deletion, as were the unaffected siblings. Both affected siblings were homozygous for all SNPs studied (Figure 4).

Sequence analysis of the 917-bp PCR product showed it did not contain exon 8. The PCR product contained unique DNA from both intron 7 and 8 at each end and included a complete Alu Y repeat in its center. By blasting this PCR product sequence, we identified that 664 bp were identical to the DNA upstream from primer 8R5 (located in intron 8). The first 389 bp of this was unique to intron 8, with a complete Alu Y repeat making up the remainder of the homology. The remaining 253 bp of the PCR product was unique to intron 7. Downstream from this 253 bp in genomic sequence is another Alu Y repeat, suggesting nonhomologous recombination between repeats as a possible cause for the deletion.

Screening of 100 unrelated probands with autosomal recessive retinal dystrophies collected in the UK, using the PCR described above, failed to identify a similar deletion. Screening of 100 probands with RP from Saudi Arabia identified a second family with recessive RP with the same exon 8 deletion. Affected members of the family were homozygous for the deletion. Haplotype analysis showed that these two families shared a region of at least 500 kb based on the two makers D2S160 and D2S1896 (chr2:112,309,575–112,815,205). This deletion was not seen in 100 control DNA samples from Saudi Arabia. This additional family was not available for detailed phenotyping. A review of the clinical records showed that there were five affected individuals with childhood onset RP. Visual acuity ranged from 20/50 in the first decade to hand movements by the third decade. There was macular atrophy present from the first decade and extensive peripheral atrophy and pigmentation.

### Screening of *MERTK* in Leber congenital amaurosis patients

Screening 292 probands with either LCA or childhood onset retinal dystrophy, using the Asper LCA mutation chip [15], identified a single patient with a known *MERTK* mutation. Analysis of the LCA chip data of subject 3 revealed the presence of the known mutation p.R651X caused by a C to T transition in exon 14 [5]. This was the only sequence variant identified by the chip. Direct sequencing of the gene in this patient confirmed the p.R651X change and also revealed a G to A sequence change in the first base of intron 1 (c.61+1G>A). This novel change is predicted to disrupt the donor splice site of intron 1. Analysis of this novel change using an automated splice site analysis predictor program [20] revealed that the mutation would eradicate the use of the true donor splice site, with the cellular splicing mechanism favoring the use of potential splice sites further downstream. A site 33 bp downstream from the mutation was predicted to be used. The extra 33 bp in exon 1 would not disrupt the frame but would insert 11 new codons into the transcript. Other potential splice sites (4 bp and 24 bp downstream from the true site) would cause a change of the reading frame leading to a stop codon in exon 2. These two mutations were shown to have been inherited from each parent and were not seen in 100 ethnically matched controls (data not shown).

## Discussion

Mutations in *MERTK* are a rare cause of retinal dystrophy in humans. The present report describes two novel disease-causing mutations identified in a large cohort of children with LCA and childhood onset rod–cone dystrophy. We identified a large deletion in *MERTK* affecting members of a consanguineous family with ARRP. Linkage was established to the *MERTK* region by a genome-wide scan using an Affymetrix SNP chip. Direct sequencing of the coding exons did not identify a single base change, but long-range PCR revealed a 9-kb deletion resulting in the removal of exon 8. The deletion occurred between two Alu Y repeats. Alu repeats are characterized by sequences approximately 300 bp long with a poly (A) tract of variable length and flanking direct repeats. Over one-third of the human genome contains repetitive interspersed sequences that were once transposable elements. Alu sequences are the second most common element (after LINE-1 elements), representing more than 5% of the genome [21].

Analysis of the *MERTK* locus identified a total of ten Alu Y repeats; nonhomologous recombination involving other Alu Y repeats could therefore be involved in other *MERTK*-associated retinal dystrophies. Large deletions in disease genes are susceptible to being missed when using direct DNA sequencing, especially if they are in an individual who is a compound heterozygote. Nonhomologous recombination between Alu repeats is a frequent cause of deletions and insertions in the human genome [22]. Such recombination events have been associated with inherited disease in several cases, including eye disorders, such as X-linked retinoschisis [23] and retinitis punctata albescens [24].

Screening of patients with LCA and childhood onset retinal dystrophies using the Asper Ophthalmics LCA chip allowed us to identify a further patient with a previously reported *MERTK* mutation. The Asper Ophthalmics LCA chip [14,15] contains known mutations and polymorphisms from LCA genes but has a limited number of variants in *MERTK*. Direct sequencing of coding exons revealed a splice site mutation in the first base of intron 1. In silico analysis using a splice site analysis predictor program suggested that this would reduce the effectiveness of the donor splice site. The next strongest potential site, if used, would introduce 11 amino acids into the protein but would not disrupt the frame of the active protein kinase domain. The other potential sites would lead to a premature stop codon in exon 2, thus eliminating most of the protein’s active domains and thus rendering it inactive. As this is early in the transcript, we predicted that these altered transcripts would be degenerated through nonsense-mediated decay (NMD). NMD is a potential outcome in transcripts where an early stop codon is recognized. Only when the premature stop codon occurs in the last coding exon or the last 55 bp of the preceding exon are these transcripts protected from NMD [25]. It is possible that NMD may occur in family A as the deletion of exon 8 leads to a premature stop codon in exon 9.

To date, six families have been reported with retinal disease due to *MERTK* mutations (Table 1) [5-11]. Gal et al. [5], in their initial report of *MERTK* mutations in human disease, described three patients with *MERTK* mutations. All three individuals had a severe retinal dystrophy of childhood onset, but few details of the phenotype are given. Subsequent reports [8,10,26] included more clinical details. McHenry et al. [8] reported a single case with a severe rod–cone dystrophy symptomatic at age 3 years. By age 9 there was nystagmus, severe visual field constriction, and an undetectable ERG.

**Table 1:** Comparison of published *MERTK* phenotypes.

Mutation	Age of Onset	Age at Exam	VA in LogMAR	Visual fields	Fundus	OCT	ERG	Ref
Hom c.2070_2074 delAGGAC	Early childhood	45	NP	Small island of remaining central vision	NP	NP	NP	[5,11]
Hom c.1605-2A>G	Early childhood	34	NP	Small island of remaining central vision	NP	NP	NP	
Het p.R651X No second mutation detected	12	12, 17	NP	Abnormally reduced (age 12)	Atrophic retinal lesions; Vessels not particular attenuated (age 17)	NP	Undetectable rod ERG (age 12)	
Het p.R722X Het p.R844C	3	9, 13	0.5 OU (age 9)	20° of central vision with peripheral crescent (age 9)	Macular atrophy, bone spicules, dense parafoveal pigmentation, heavy RPE granularity (age 9)	NP	Undetectable scotopic & photopic responses	[8]
			1.00 OU (age 13)	<5°central vision with peripheral crescent (age 13)	Bull’s eye macular atrophy, RPE thinning at periphery (age 13)			
Hom c.2214delT	12	16	0.2 OD 0.3 OS	Well preserved	Bull’s eye macular atrophy, attenuated vessels, pale reflex from the RPE	Unremarkable; normal retinal thickness	Abnormal scotopic & photopic responses, delayed 30 Hz flicker, undetectable PERG	[10]
	<10	14	0.2 OU		Bull’s eye macular atrophy, attenuated vessels, pale reflex from the RPE, crystals in the macula			
	10	10	0.0 OU		Crystals near the fovea, pale reflex from the RPE		Absent scotopic, severely attenuated photopic responses, delayed 30 Hz flicker, barely detectable pERG	
	9	9	OU	NP	NP	NP	NP	
Hom c.2189+1G>T	Early childhood	5	0.4 OD 0.5 OS	NP	White-yellowish small subretinal deposits in the macula, mildly attenuated vessels	Thinning of neurosensory retina (thin ONL & major alterations to the OLM)	Absent scotopic, severely attenuated photopic responses	[7,26]
		9	0.4 OD 0.5 OS	NP	Yellowish macular atrophy, mildly attenuated vessels, wrinkled appearance of inner retina		Undetectable scotopic & photopic responses	
		16	1.0 OD 0.4 OS	Well preserved for Goldman III4e, variable concentric constriction for I4e	Yellowish macular atrophy, mildly attenuated vessels, wrinkled appearance of inner retina, salt-like pigment mottling at the mid periphery	Similar OCT findings; sd-OCT showed debris like material in subneurosensory space		
		18	0.4 OU					
		19	0.7 OD 1.3 OS		Yellowish macular atrophy, crystalline retinal deposits, mildly attenuated vessels, wrinkled appearance of inner retina, salt-like pigment mottling at the mid periphery and bone spicules in the outer periphery of the LE	Similar OCT findings as siblings		
Hom Del of ex8	9	26	1.78 OU	NP	Macular atrophy, attenuated vessels, bone spicules in the mid periphery, pale discs	Sd-OCT showed thinning of the ONL and debris like material below the OLM	not tested	this Study
	Early childhood	8	0.32 OU	20-30° of preserved central fields	Bull’s eye macular atrophy, bone spicules and granular RPE appearance in the mid periphery		not tested	
Het p.R651X Het c.61+1G>A	12	22	0.6 OD 1.0 OS	Well preserved	Focal atrophy at macula, very little intraretinal pigment		Severely abnormal scotopic & photopic responses, barely detectable pERG	

Table showing the comparison of published *MERTK* phenotypes with patients 1, 2, and 3. NP represents not published.

Tschernutter et al. [10] reported three affected individuals from a consanguineous family from the Middle East who had a homozygous null mutation. Affected individuals reported night blindness from early childhood, with subsequent reduction in central vision. The rod ERG was severely abnormal at diagnosis with later cone involvement. The pattern ERG was abnormal early in the disease. An interesting feature of the disease was the well preserved peripheral field. Charbel Issa et al. [26] have recently reported similar findings in five affected individuals from a consanguineous Moroccan family. The two affected members of our family A have a phenotype similar to the individuals reported by Tschernutter et al. [10] and Charbel Issa et al. [26], with early onset of night blindness, early rod ERG reduction, and early macular atrophy. Our third patient had a milder overall phenotype with later onset of disease. However, he shows the typical characteristic of early macular atrophy and relative preservation of peripheral visual fields. Spectral domain OCT shows loss of photoreceptors and hyper-reflective bodies possibly analogous to the debris layer seen in the RCS rat. Similar spectral domain OCT changes were reported by Charbel Issa et al. [26]. These changes as well as a wavelike appearance of the innermost neurosensory retina might be distinctive OCT findings in patients with mutations in *MERTK*.

The phenotype associated with *MERTK* mutations is of a childhood onset rod–cone dystrophy with early macular atrophy. The OCT appearance is distinctive and may help guide targeted molecular genetic analysis for the identification of patients with *MERTK* mutations. The retinal dystrophy seen in the animal model of *MERTK*-related disease (the RCS rat) has been successfully rescued with gene replacement therapy [27]. It is evident from the findings in our patients and others reported in the literature that there is a window of opportunity for therapeutic intervention in childhood. This makes *MERTK* an excellent target for gene therapy in humans, and the early identification of patients will be important for their inclusion in future clinical trials.
